# Crucial Role of Calcium-Sensing Receptor Activation in Cardiac Injury of Diabetic Rats

**DOI:** 10.1371/journal.pone.0065147

**Published:** 2013-05-22

**Authors:** Hanping Qi, Yonggang Cao, Wei Huang, Yang Liu, Ye Wang, Lei Li, Lijuan Liu, Zhong Ji, Hongli Sun

**Affiliations:** 1 Department of Pharmacology, Harbin Medical University-Daqing, Daqing, Heilongjiang, China; 2 Department of Basic Nursing, Harbin Medical University-Daqing, Daqing, Heilongjiang, China; 3 Department of Surgery, Fifth Clinical College of Harbin Medical University, Daqing, Heilongjiang, China; 4 Department of Pathology, Harbin Medical University-Daqing, Daqing, Heilongjiang, China; 5 Department of Physiology, Harbin Medical University-Daqing, Daqing, Heilongjiang, China; Consiglio Nazionale delle Ricerche, Italy

## Abstract

Cardiac injury is a common pathological change frequently accompanied by diabetes mellitus. Recently, some evidence indicated that calcium-sensing receptor (CaSR) expressed in the cardiac tissue. However, the functional role of CaSR in diabetic cardiac injury remains unclear. The present study was designed to investigate the relationship between CaSR activation and diabetes-induced cardiac injury. Diabetic model was successfully established by administration of streptozotocin (STZ) *in vivo*, and cardiomyocyte injury was simulated by 25.5 mM glucose *in vitro*. Apoptotic rate, intracellular calcium concentration ([Ca^2+^]_i_) and the expression of Bcl-2, Bax, extracellular signal-regulated protein kinase (ERK), c-Jun NH_2_-terminal protein kinase (JNK), and p38 were examined. We demonstrated a significant increase in left ventricular end-diastolic pressure (LVEDP) as well as decrease in maximum rate of left ventricular pressure rise and fall (±dp/dtmax), and left ventricular systolic pressure (LVSP), apoptosis of cardiomyocytes was also observed by TUNEL staining. *In vitro*, 25.5 mM glucose-induced apoptosis was detected by flow cytometry in neonatal rat cardiomyocytes. Further results showed that 25.5 mM glucose significantly increased [Ca^2+^]_i_, up-regulated the expression of Bax, P-ERK and P-JNK, and suppressed Bcl-2 expression. However, the above deleterious changes were further confirmed when co-treatment with CaSR agonist GdCl_3_ (300 µM). But the effects of GdCl_3_ were attenuated by 10 µM NPS-2390, a specific CaSR inhibitor. When CaSR was silence by siRNA transfection, the effects of high glucose were inhibited. These results suggest that CaSR activation could lead to the apoptosis of cardiomyocytes in diabetic cardiac injury through the induction of calcium overload, the activation of the mitochondrial, and mitogen-activated protein kinase pathway.

## Introduction

The incidence of diabetes mellitus is increasing worldwide and one of common pathological change is cardiac injury. In the clinic, two-thirds of the diabetic patients die from cardiovascular diseases [1]. Furthermore, the risk of heart failure was increased in diabetic men and women by two- and five folds, respectively [2]. Therefore, diabetes-induced cardiac injury has become a major cause of diabetes related morbidity and mortality. Accumulating evidences indicated that the apoptosis has been recognized as a factor of diabetes-induced cardiac injury. However, the pathogenic mechanisms that apoptosis is involved in diabetes-induced cardiac injury remain unclear. Several reports have suggested that increasing intracellular Ca^2+^ contributes to the development and progression of cardiomyocyte apoptosis.

The calcium-sensing receptor (CaSR) belongs to a member of G-protein coupled receptor and is organized into three major structural domains: a large amino-terminal in extracellular domain (ECD), the typical seven transmembrane domains (TMD), and a cytoplasmic carboxyterminal [3]. CaSR was cloned originally from parathyroid chief cells in 1993 [4]. The existence of CaSR has also been identified in thyroid [5], kidney [6], bone [7], and gastrointestinal tract tissues [8], which participates in the regulation of systemic calcium homeostasis. In 2003 [9], we first reported the expression of CaSR in cardiac tissue and also revealed that the activation of the CaSR lead to intracellular calcium release via G protein-phospholipase C (PLC)-inositol, 4, 5-triphosphate (IP_3_) pathway. The increase in intracellular calcium concentration ([Ca^2+^]_i_) through CaSR could be involved in many cell activities, such as cell proliferation and apoptosis. During cardiac ischemia/reperfusion (I/R) and cardiac hypertrophy, we found that CaSR expression increased and was involved in calcium overload-induced cardiomyocyte apoptosis [10], [11]. However, the expression and function of CaSR in diabetes-induced cardiac injury are poorly understood.

Our study showed that CaSR protein level increased in rat ventricular tissue after two months of streptozotocin administration. Therefore, we speculated that the CaSR could be directly or indirectly involved in diabetes-induced cardiac injury. However, the effects and mechanisms of CaSR on experimental diabetic cardiac injury are not well characterized. Therefore, the aim of the present study was to evaluate the involvement of CaSR in diabetes-induced cardiac injury and to explore possible mechanism.

## Materials and Methods

### Ethics statement

All experimental protocols were pre-approved by the Experimental Animal Ethic Committee of Harbin Medical University, China (Animal Experimental Ethical Inspection Protocol No. 2009104). Use of animals was confirmed with the Guide for the Care and Use of Laboratory Animals published by the US National Institutes of Health (NIH Publication No. 85–23, revised 1996).

### Induction of experimental diabetes

Diabetes mellitus was induced in Wistar rats (200–250 g, provided by the Experimental Animal Center of Harbin Medical University, Grade II) with a single intraperitoneal injection (i.p.) of 40 mg/kg streptozotocin (STZ, Sigma Chemical Co., USA), freshly prepared solution in 0.1 M citrate buffer (pH 4.5). Blood sample was collected from tail vein and blood glucose was measured 3 days later after STZ. Plasma glucose levels ≥16.7 mM (2 times continuously) were considered to be a successful rat model with diabetes. The diabetic rats were randomly divided into four groups 2 months after STZ: (1) model group; (2) model+GdCl_3_ group: diabetic rats were injected GdCl_3_ (agonist of CaSR, Sigma Chemical Co., USA) at 8.67 mg/kg for 1 week; (3) model+NPS-2390+GdCl_3_ group: 0.20 g/kg NPS-2390 (antagonist of CaSR, Sigma Chemical Co., USA) was administered 2 h of re-injection of GdCl_3_ for 1 week; (4) model+NPS-2390 group: 0.20 g/kg NPS-2390 was used for 1 week. The control group only received an injection of same amount of citrate buffer, and additional GdCl_3_ (8.67 mg/kg) was added in control+GdCl_3_ group.

### Hemodynamic measurements

After 2 months of diabetic rat model was established, under the anesthesia with pentobarbital sodium (40 mg/kg), a heparin-filled cannula was inserted into the right carotid artery and then advanced into the left ventricle, where the hemodynamic parameters reflecting cardiac performances, such as left ventricular systolic pressure (LVSP), left ventricular end-diastolic pressure (LVEDP), and maximum rate of left ventricular pressure rise and fall (+dp/dtmax and -dp/dtmax) were measured by a pressure transducer interfaced with BL-420E organism function experiment system (Cheng Du Tai Meng, China). In the condition of anesthetization with pentobarbital sodium (40 mg/kg), the rat was sacrificed by cervical dislocation and the heart was removed rapidly and washed with ice-cold 0.9% saline after the hemodynamic measurements had been obtained. Parts of the heart were frozen in liquid nitrogen or fixed in 4% paraformalin for later use.

### TUNEL staining

Terminal deoxynucleotidyl transferase-mediated dUTP nick end labeling (TUNEL) assay [12], [13] was used to assess myocardial apoptosis with an apoptosis detection kit (Roche, Basel, Switzerland). TUNEL-positive cardiomyocytes in the each group were carefully evaluated under double-blind conditions. 10 high-power fields (*×*400) were randomly selected, and the percentage of TUNEL-positive cells was determined by dividing the numbers of positive-staining nuclei by the numbers of total nuclei of the cells.

### Neonatal rat cardiomyocytes incubation

Neonatal rat cardiomyocytes were prepared from 2- to 3-day-old neonatal Wistar rats. The neonatal rats were anesthetized with dry ice (CO_2_), and then immersed in 70% (v/v) ethanol. The ventricles were removed and washed 3 times with D-Hanks balanced salt solution at 4°C, then minced and incubated with 0.25% (w/v) trypsinase at 37°C for 10 min. Addition of an equal volume of cold Dulbecco's modified Eagle's medium (DMEM) containing 10% (v/v) fetal bovine serum (FBS, Sigma Chemical Co., USA) was used to terminate the digestion. The supernatant was discarded. The latter digestion step was repeated 4 times. Cells in the supernatant were isolated by centrifugation at 1500 rev./min at room temperature for 10 min. Cells were re-suspended in DMEM containing 20% (v/v) FBS, 100 U/mL penicillin and 100 mg/mL streptomycin, and then were cultured as monolayers at a density of 5×10^4^ cells/cm^2^ at 37°C in a humidified atmosphere containing 5% (v/v) CO_2_. Neonatal rat cardiomyocytes were randomly divided into six groups: (1) control group; (2) model group: cells were incubated with 25.5 mM glucose for 24 h; (3) model+GdCl3 group: cells were given 300 µM GdCl3 for 4 h after treatment with 25.5 mM glucose for 24 h; (4) model+NPS-2390+GdCl3 group: 10 µM NPS-2390 was administered for 2 h before exposure to 300 µM GdCl3, following 24 h treatment with 25.5 mM glucose; (5) model+NPS-2390 group: 10 µM NPS-2390 was added for 2 h after 24 h treatment with 25.5 mM glucose; (6) control+GdCl3 group: cells were given 300 µM GdCl3 for 4 h.

### siRNA transfection

CaSR was silenced by using a small interfering RNA (siRNA) targeting rat CaSR with the following sequence: 5′-GCCUGAGUAUUUCCAUGUATT-3′. This siRNA which was purchased from Gene Pharma (Shanghai, China) was annealed according to manufacturer's instructions and then stored at −20°C. Cardiomyocytes were transfected with siRNA (1.0 µg/mL) using lipofectamine 2000 according to the manufacturer's instruction and incubated at 37°C for 48 h. Transfected cells were randomly divided into three groups: (1) siRNA+model group: cells were transfected with 1.0 µg/mL siRNA for 48 h, then were incubated with 25.5 mM glucose for 24 h; (2) siRNA+model+GdCl3 group: cardiomyocytes transfected with siRNA were given 300 µM GdCl3 for 4 h after treatment with 25.5 mM glucose for 24 h; (3) nRNA+model group: cells were transfected with 1.0 µg/mL of non-silencing siRNA (5′-UUCUCCGAACGUGUCACGUTT-3′) which is ineffective in rat cells since it has no mammalian target for 48 h, then were incubated with 25.5 mM glucose for 24 h.

### Flow cytometric analysis

The apoptosis rate of neonatal rat cardiomyocytes was detected by flow cytometric analysis [14], [15]. Both attached and floating cells were harvested for cell cycle analysis. For early apoptosis, attached cells were harvested for sorting in annexin-V-fluorescein and PI buffer (Roche, Nonnenwald, Penzberg, Germany). For each experiment, 10,000 cells were analyzed using ELITE Flow Cytometry (BD Biosciences, San Jose, CA) and Cell Quest software (BD Biosciences). Triplicates were performed in all cases except for the detection of early apoptotic cells.

### Western blot analysis

Total proteins of the neonatal rat cardiomyocytes were prepared and quantified using the BCA Protein Assay Reagent. Protein from different experimental groups were separated by electrophoresis on 10% SDS-PAGE and transferred to PVDF membranes. The membranes were blocked with 5% non-fat milk at room temperature for 2 h. The membranes were then incubated overnight at 4°C with antibody against CaSR (1∶2000), anti-JNK (1∶800), anti-ERK1/2 (1∶800), and anti-p38 (1∶500), respectively. The membranes were incubated with secondary antibody for 1 h at room temperature in the dark. The membrane was then washed 3 times with PBS and scanned by an Odyssey infrared imaging system (LI-COR, Lincoln, NB) at a wave length of 800 nm.

### Measurement of [Ca^2+^]_i_

Fluorescence measurements in cardiomyocytes have been described previously [16]. The neonatal rat cardiomyocytes were incubated with a working solution containing 10 µM Fluo-3/AM (acetoxymethyl ester form, Molecular Probes) and 0.03% Pluronic F-127 at 37°C for 40 min. Then, the cardiomyocytes were rinsed twice with Tyrode solution to remove the remaining dye. The changes of [Ca^2+^]_i_ were represented as fluorescent intensity (FI). During the experiment, FI of Fluo-3/AM in cardiomyocytes was detected for 5 min using a laser scanning confocal microscope (Olympus, Japan) with excitation at 488 nm and emission at 530 nm. FI was observed in ten randomly chosen cells to calculate the average FI for all cells.

### Statistical analysis

All data were expressed as mean±SEM and were analyzed using SPSS 15.0 software. Statistical comparisons among multiple groups were performed by analysis of variance (ANOVA). If significant effects were indicated by ANOVA, SNK-q (Student-Newman-Keuls) test was used to evaluate the significance of differences between individual means. *P*<0.05 was considered to be a statistically significant difference.

## Results

### Changes of plasma glucose and body weight in diabetic rats

**Figure 1:**
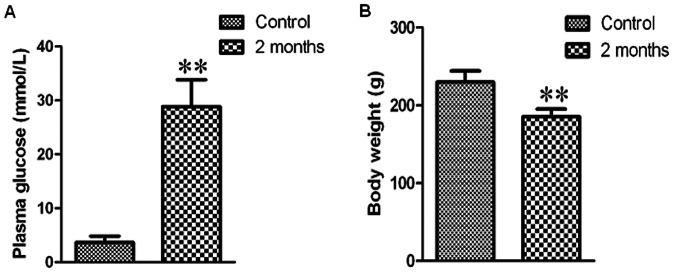
Changes of plasma glucose and body weight in diabetic rats. Bar graph of plasma glucose (A) and body weight (B). Values were expressed as mean±SEM (*n* = 8). **P<0.01 compared with control group.

Diabetic rats were induced by intraperitoneal injection of STZ (40 mg/kg) in our experimental condition. As shown in Fig. 1, the blood glucose levels were significantly increased and the body weight was obviously decreased in model group (*P<*0.01) compared with control group. The results indicated that diabetic rat model was established successfully.

### Expression of CaSR in diabetic rat hearts

**Figure 2:**
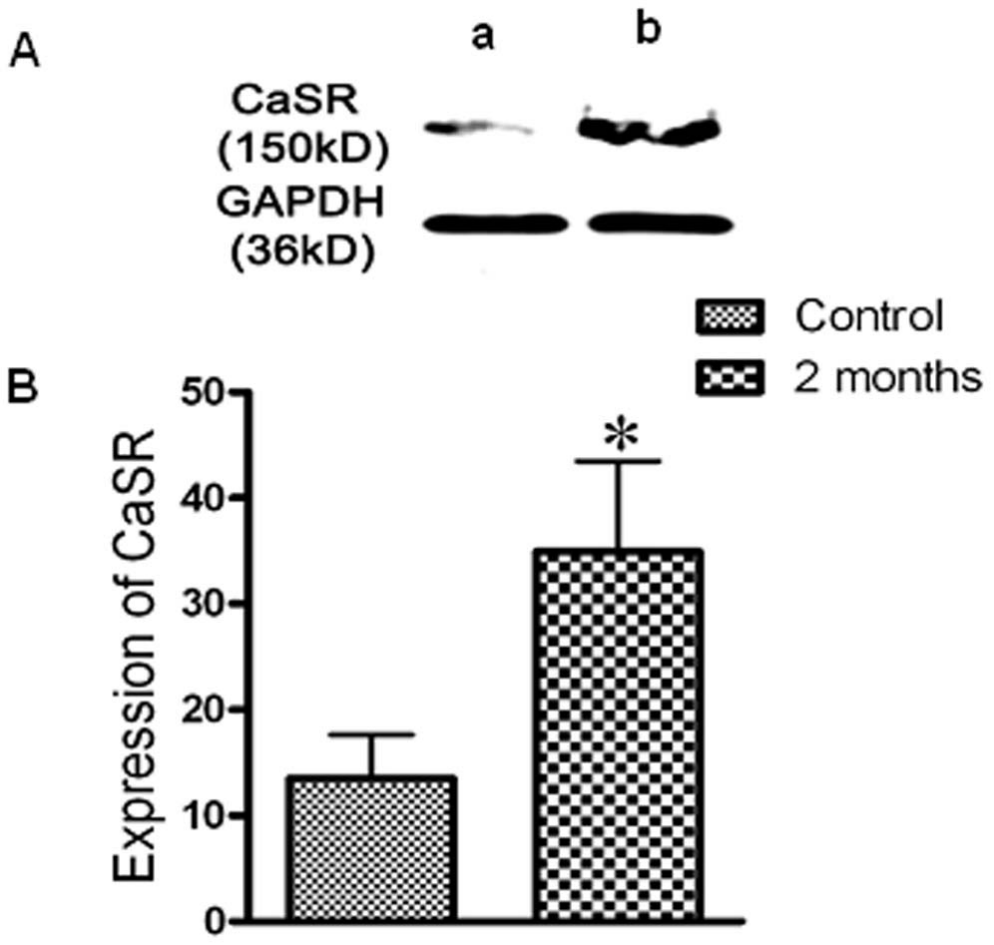
CaSR protein expression determined by western blot analysis in diabetic rat hearts. **(**A) Representative bands of CaSR in diabetic rat hearts. a, control; b, two months. (B) The data represent the mean±SEM of eight independent experiments. The intensity of each band was quantified using densitometry, and the data were normalized to band intensity of GAPDH used as internal control. *P<0.05 compared with control group.

After the model of diabetes mellitus was established, rats were sacrificed and ventricular tissues were prepared to assess the CaSR protein expression after two months of STZ administration. In the western blot analysis, two bands corresponding to the CaSR with a relative molecular mass of 150 kD and 170–180 kD were detected. The CaSR protein level in the two months rats was higher than that in the control group (*P<*0.05) (Fig. 2).

### Effects of CaSR on hemodynamics in diabetic rat hearts

**Figure 3:**
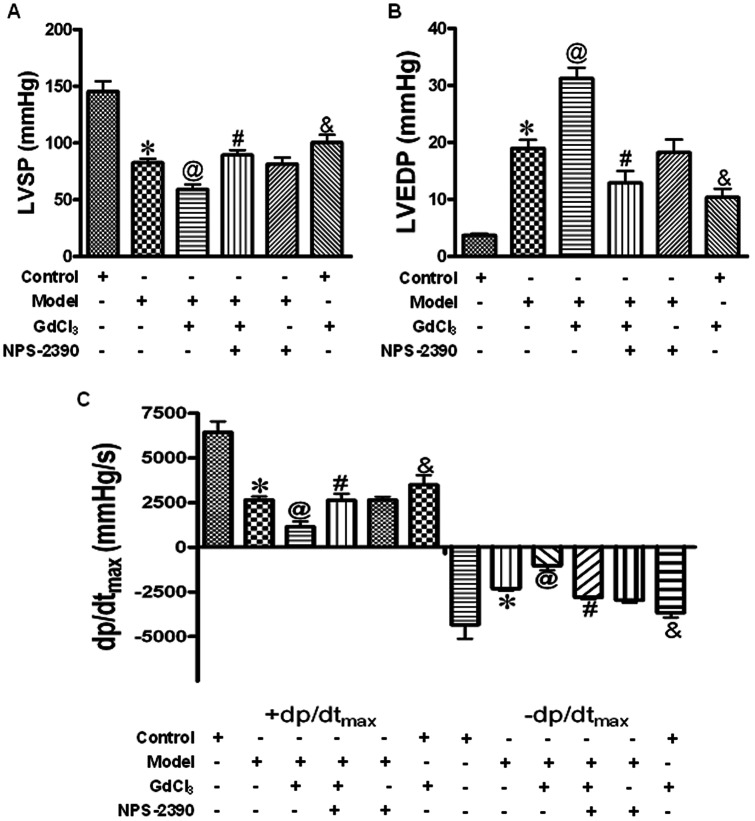
Effects of CaSR on hemodynamics in diabetic rat hearts. The hemodynamic parameters, LVSP (A), LVEDP (B), +dp/dtmax and -dp/dtmax (C) were measured throughout the experiment. Data were represented by mean±SEM (*n* = 8 per group). *P<0.05 compared with control group; @*P*<0.05 compared with model group; #*P*<0.05 compared with model+GdCl3 group; &*P*<0.05 compared with control group.

To examine the effects of CaSR on cardiac function, ventricular hemodynamics was monitored after two months of STZ injection. As shown in Fig. 3, there were significant increases in LVEDP as well as reduction in ±dp/dtmax and LVSP in model group (compared with control group, *P*<0.05), indicating that the cardiac dysfunction was developed in all diabetic model rats. In the GdCl_3_ group, the abnormal hemodynamic parameters were further aggravated (*P*<0.05), but these deleterious changes can be attenuated by co-treatment with NPS-2390 and GdCl_3_ (*P*<0.05).

### Effects of CaSR on cardiomyocyte apoptosis in diabetic rats by TUNEL staining

**Figure 4:**
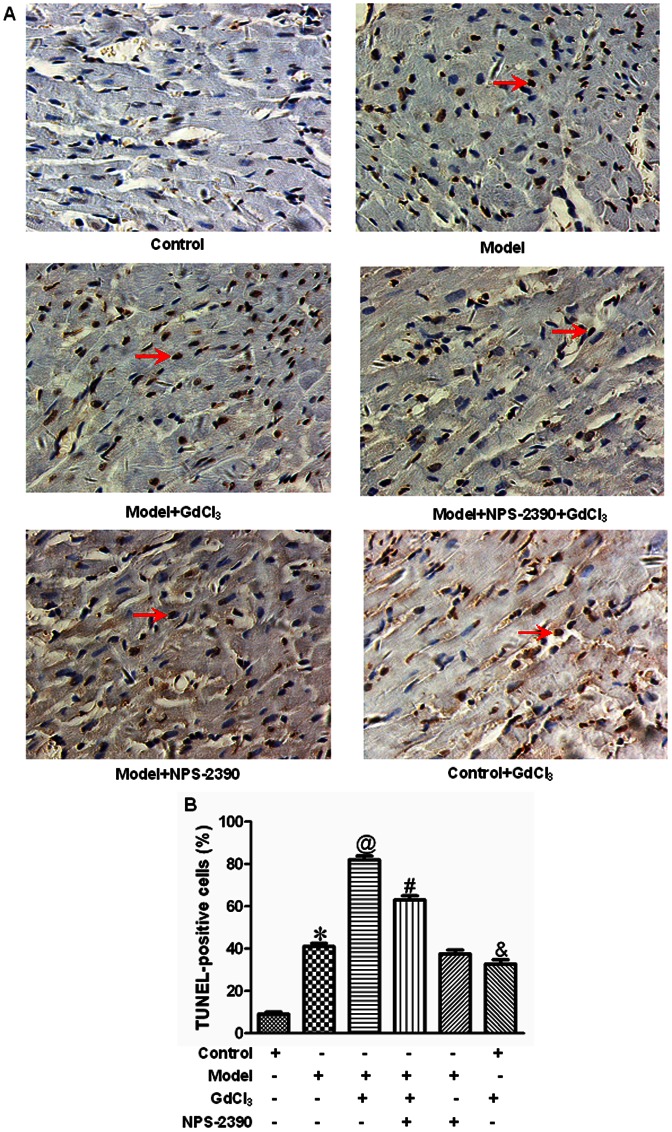
Representative illustration of TUNEL staining in cardiomyocytes apoptosis. (A) Nuclei with brown staining were TUNEL-positive cell, which was defined as apoptotic cell. Magnification at 400 ×. (B) Statistical analysis of cardiomyocytes apoptosis (*n* = 8). *P<0.05 compared with control group; @*P*<0.05 compared with model group; #*P*<0.05 compared with model+GdCl3 group; &*P*<0.05 compared with control group.

To explore whether the activation of CaSR was related to diabetes-induced cardiomyocyte apoptosis, we used TUNEL staining to locate apoptotic nuclei, and found that low level of TUNEL-positive cells were observed in the cardiac muscle of rats in control group, whereas a large number of TUNEL-positive cells were detected in the rats subject to STZ for two months. Compared with model group, the number of TUNEL-positive cells was significantly increased in the GdCl_3_ group (*P*<0.05). The effects of GdCl_3_ on cell apoptosis could be improved by NPS-2390 (*P*<0.05) (Fig. 4).

### Simulation of myocardial injury in neonatal rat cardiomyocytes by exposure to high concentration of glucose

**Figure 5:**
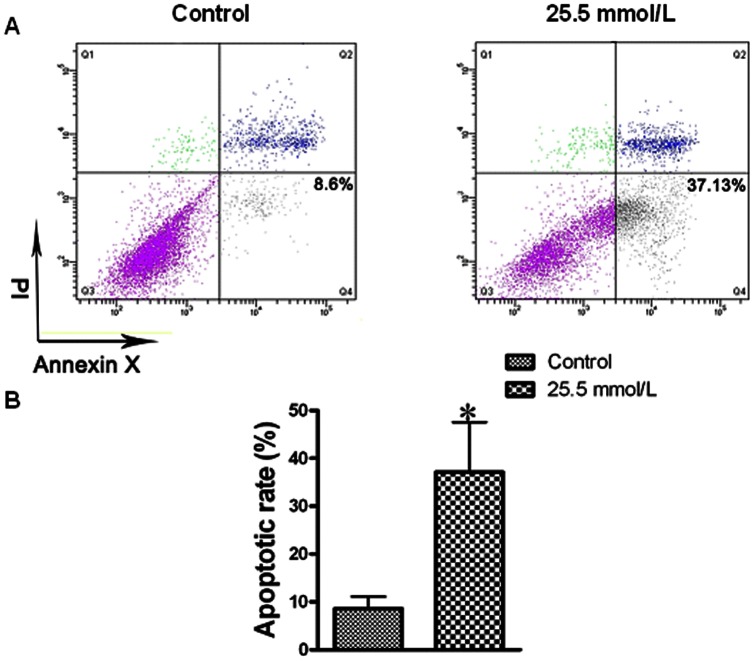
Flow cytometric analysis of apoptosis. A, 10,000 cells of each experiment were collected by flow cytometry and analyzed using Cell Quest software. Early apoptotic cells (annexin-V+ and PI-) were displayed in the lower right quadrant, and late apoptotic cells (annexin-V+ and PI+) were shown in the upper right quadrant. (A) Plots of sorted apoptotic cells. Apoptosis were evaluated after treating neonatal rat cardiomyocytes with 25.5 mM glucose, and staining with annexin-V and PI. Flow cytometry profile represents annexin-V-FITC staining in *x* axis and PI in *y* axis. The number represents the percentage of apoptotic cells in each condition. (B) Bar graph of cell apoptotic rate. All data were expressed as mean±SEM (*n* = 6). *P<0.05 compared with control group.

Metabolic changes in diabetes can be directly triggered by hyperglycemia. Diabetic hearts have primary defect in the stimulation of glycolysis and glucose oxidation. Altered substrate supply and utilization by cardiomyocytes could be the primary injury in the pathogenesis of diabetic cardiomyopathy. So we used hyperglycemia to simulate diabetic cardiomyopathy in neonatal rat cardiomyocytes. Flow cytometry was used to examine the percentage of cardiomyocyte apoptosis. Compared with control value, the 25.5 mM level of glucose significantly increased the apoptotic rate of cardiomyocytes (Fig. 5).

### Effects of CaSR on cell apoptosis in neonatal rat cardiomyocytes exposed to high concentration of glucose by flow cytometry

**Figure 6:**
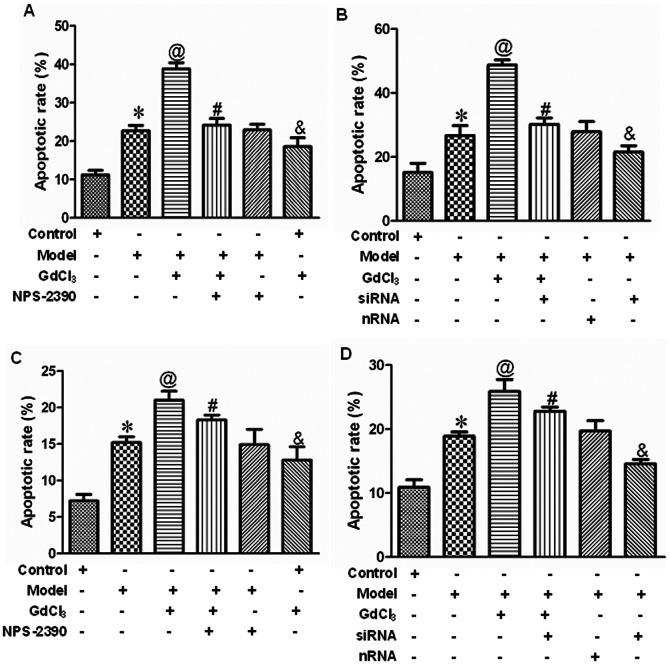
Detection of apoptosis in neonatal rat cardiomyocytes by flow cytometry. Bar graph of early (A and B) and late (C and D) apoptotic rate. All data were expressed as mean±SEM (*n* = 6). *P<0.05 compared with control group; @*P*<0.05 compared with model group; #*P*<0.05 compared with model+GdCl3 group; &*P*<0.05 compared with control group. Groups in A and C were control, model, model+GdCl3, model+NPS-2390+GdCl3, model+NPS-2390, and control+GdCl3. Groups in B and D were control, model, model+GdCl3, siRNA+model+GdCl3, nRNA+model, and siRNA+model. Glucose: 25.5 mM; GdCl3: 300 µM; NPS-2390: 10 µM.

The cardiomyocyte stained with both annexin-V and PI can be used as a specific marker for apoptosis in the early phase where the cell membrane is still intact. The results showed that early apoptosis and late apoptosis were elevated gradually after application of 25.5 mM level of glucose for 24 h compared with that in control group (*P*<0.05). GdCl_3_ further strengthened the apoptotic rate (*P*<0.05, compared with model group). Moreover, pretreatment with NPS-2390, a selective antagonist of CaSR, lessened the impaired effect of GdCl_3_ (*P*<0.05) (Fig. 6A and 6C). When the CaSR was silenced by siRNA technique, the apoptotic rate was reduced compared with model group (Fig. 6B and 6D). Based on above results, it is speculated that CaSR is involved in high glucose-induced cardiomyocyte apoptosis.

### Effects of CaSR on [Ca^2+^]_i_ in neonatal rat cardiomyocytes exposed to high concentration of glucose

**Figure 7:**
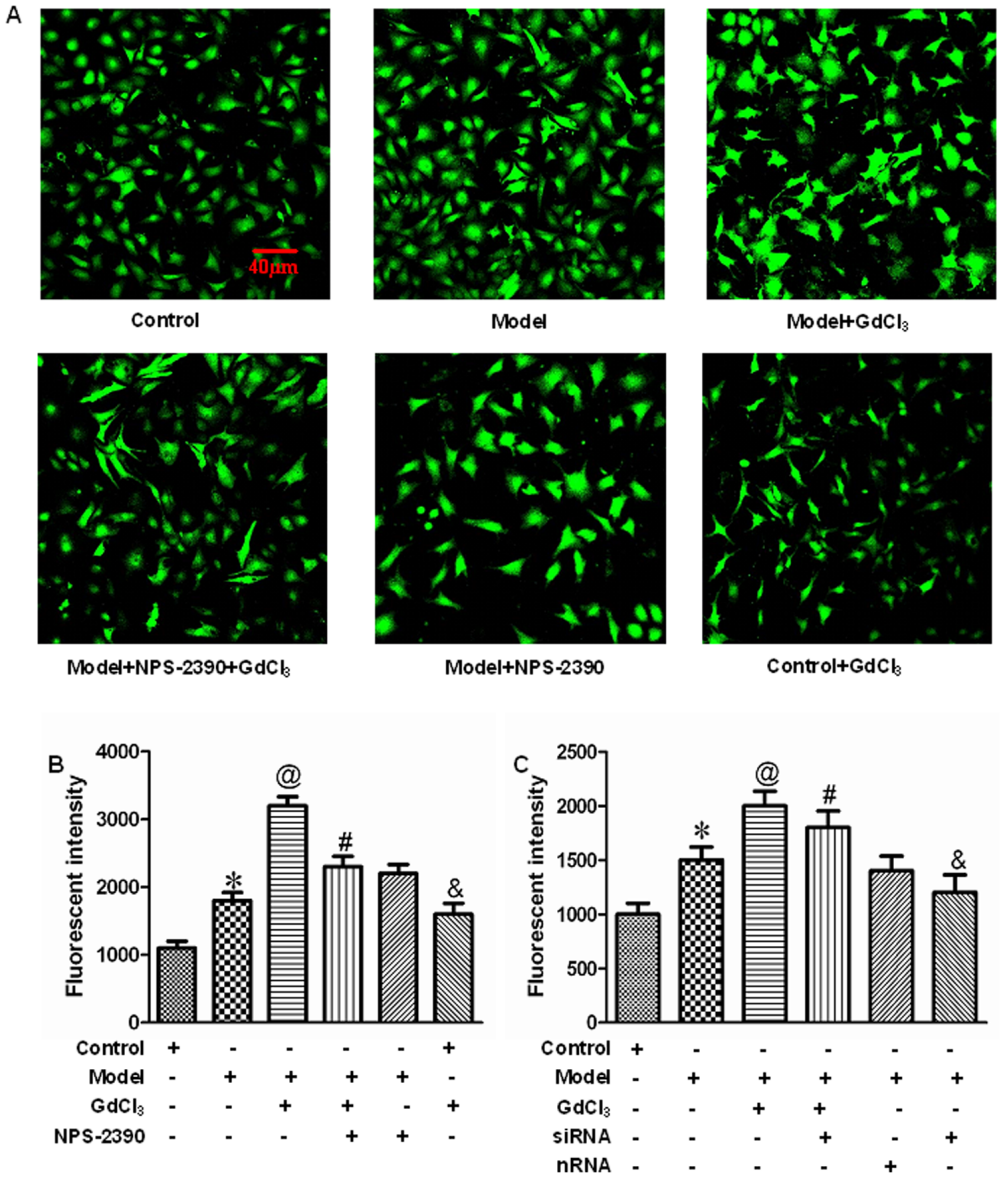
Measurement of [Ca^2+^]_i_ in neonatal rat cardiomyocytes. Fluorescent intensity in [Ca2+]i was recorded by laser scanning confocal microscope in different treatment. (A) Representative images of cardiomyocytes. (B and C) Bar graph of fluorescent intensity. All data were expressed as mean±SEM (*n* = 6). *P<0.05 compared with control group; @*P*<0.05 compared with model group; #*P*<0.05 compared with model+GdCl3 group; &*P*<0.05 compared with control group. Groups in B were control, model, model+GdCl3, model+NPS-2390+GdCl3, model+NPS-2390, and control+GdCl3. Groups in C were control, model, model+GdCl3, siRNA+model+GdCl3, nRNA+model, and siRNA+model. Glucose: 25.5 mM; GdCl3: 300 µM; NPS-2390: 10 µM.

In the present study, we investigated whether activation of CaSR could induce changes of [Ca2+]i in high glucose-treated cardiomyocytes. From fluorescence intensity taken by laser scanning confocal microscope, we found that high concentration of glucose remarkably increased [Ca2+]i (*P*<0.05), and this increase in [Ca2+]i was further enhanced by GdCl3 (*P*<0.05). However, this effect of GdCl_3_ was attenuated by NPS-2390 (*P*<0.05) (Fig. 7B). When silencing the CaSR, [Ca2+]i was decreased compared with model group (*P*<0.05) (Fig. 7C).

### Effects of CaSR on the expression of Bcl-2 and Bax protein in neonatal rat cardiomyocytes exposed to high concentration of glucose

**Figure 8:**
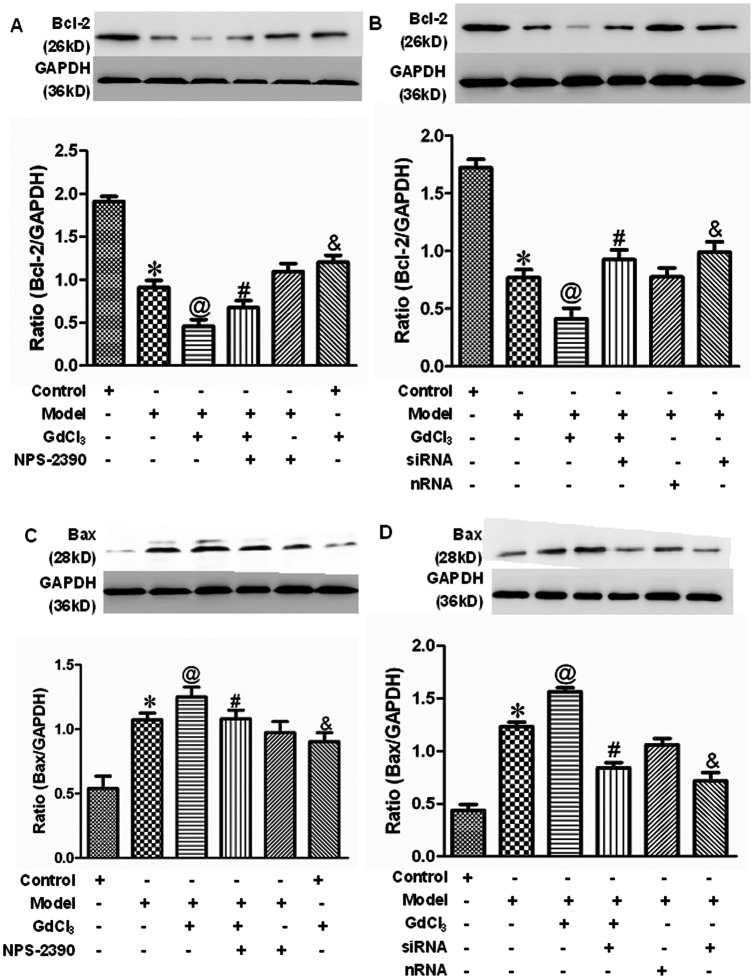
Detection of Bcl-2 and Bax protein expression in cardiomyocytes by western blot analysis. (A and B) Western blot assay for Bcl-2 expression in neonatal rat cardiomyocytes. (C and D) Western blot assay for Bax expression in neonatal rat cardiomyocytes. Average data were represented by mean±SEM (*n* = 6). *P<0.05 compared with control group; @*P*<0.05 compared with model group; #*P*<0.05 compared with model+GdCl3 group; &*P*<0.05 compared with control group. Groups in A and C were control, model, model+GdCl3, model+NPS-2390+GdCl3, model+NPS-2390, and control+GdCl3. Groups in B and D were control, model, model+GdCl3, siRNA+model+GdCl3, nRNA+model, and siRNA+model. Glucose: 25.5 mM; GdCl3: 300 µM; NPS-2390: 10 µM.

To investigate the relationship between CaSR activation and apoptosis pathway, we analyzed the expression of anti-apoptotic Bcl-2 and pro-apoptotic Bax by western blot. The present data indicated that the Bcl-2 protein level was decreased after treatment with 25.5 mM glucose (*P*<0.05), and GdCl3 further down-regulated the Bcl-2 expression while it was up-regulated in the presence of NPS-2390 on top of GdCl3 (Fig. 8A). When the CaSR was silenced by siRNA technique, Bcl-2 expression was increased compared with model group (*P*<0.05) (Fig. 8B). The opposite trend presented in the expression of Bax (Fig. 8C and 8D).

### Effects of CaSR on the expression of ERK1/2, JNK and p38 protein in neonatal rat cardiomyocytes exposed to high concentration of glucose

**Figure 9:**
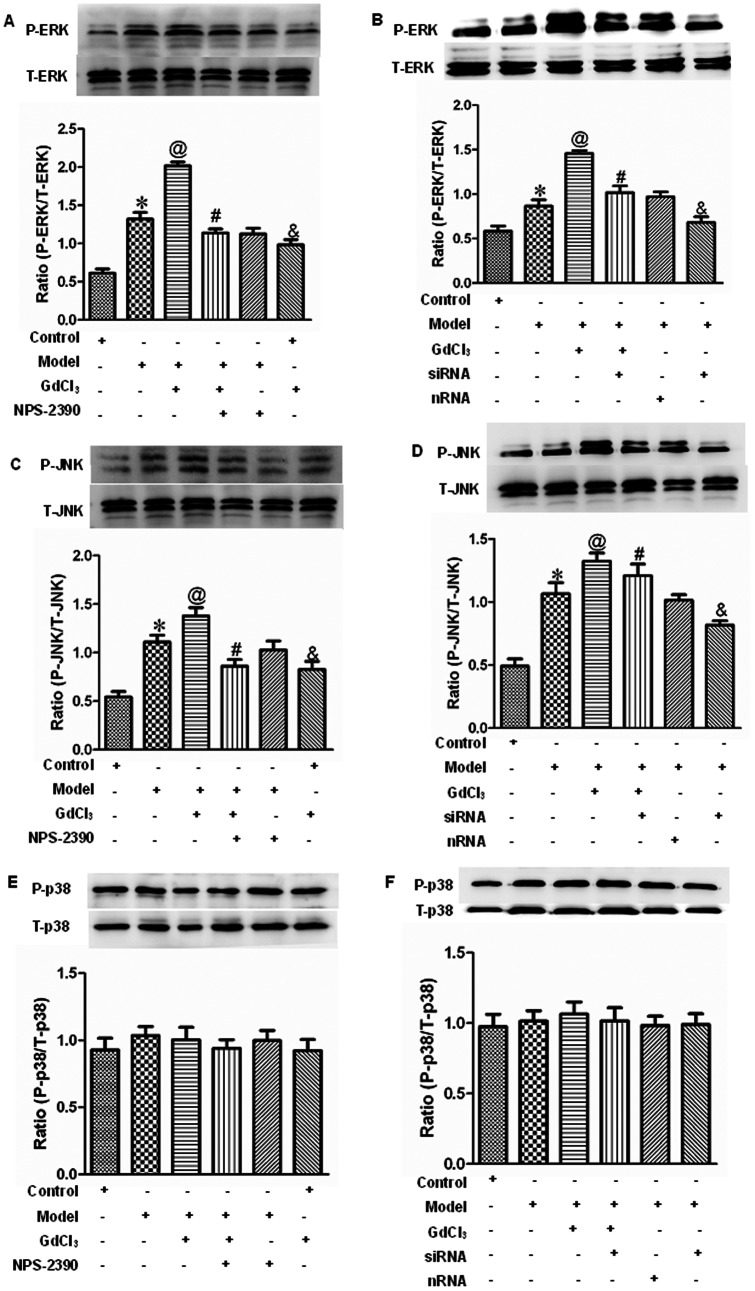
ERK1/2, JNK, and p38 phosphorylation in neonatal rat myocytes in different groups. Western blotting for phosphorylated ERK1/2 (A and B), JNK (C and D), p38 (E and F), as well as total ERK1/2, JNK, p38 of cardiomyocytes. All the data were expressed as mean±SEM (*n* = 6). *P<0.05 compared with control group; @*P*<0.05 compared with model group; #*P*<0.05 compared with model+GdCl3 group; &*P*<0.05 compared with control group. Groups in A, C and E were control, model, model+GdCl3, model+NPS-2390+GdCl3, model+NPS-2390, and control+GdCl3. Groups in B, D and F were control, model, model+GdCl3, siRNA+model+GdCl3, nRNA+model, and siRNA+model. Glucose: 25.5 mM; GdCl3: 300 µM; NPS-2390: 10 µM.

The extracellular signal-regulated protein kinase (ERK), c-Jun NH_2_-terminal protein kinase (JNK), and p38 play important roles in cell proliferation and apoptosis. To determine whether CaSR-induced apoptosis through the mitogen-activated protein kinase signal pathway, ERK1/2, JNK, and p38 were analyzed by western blot. The results showed that the total ERK1/2, JNK, and p38 were equivalent in the different groups. When the cells were exposed to glucose, P-ERK1/2 and P-JNK expressions were up-regulated (*P*<0.05), which were further enhanced by GdCl3 and reduced by NPS-2390 (*P*<0.05) (Fig. 9A and 9C). When the CaSR was silenced by siRNA technique, P-ERK1/2 and P-JNK expressions were decreased compared with model group (*P*<0.05) (Fig. 9B and 9D). Moreover, there was no effect on the expression of P-p38 (Fig. 9E and 9F).

## Discussion

Diabetes-induced cardiac injury is structural changes in hearts independent of hypertension, coronary artery disease, and valvular heart diseases. With the development of disease, there are increases in myocardial apoptosis and fibrosis [17], [18]. However, very little is known about the role of CaSR in diabetes-induced cardiac apoptosis as yet. In the present study, we used GdCl3 (a specific CaSR activator), NPS-2390 (a specific CaSR antagonist) and silencing of CaSR by siRNA techniques to examine whether CaSR activation was involved in cardiomyocytes apoptosis in experimental diabetic models.

The results showed that administration of STZ for 2 months led to the increase in the expression of CaSR at protein level in hearts of diabetic model rats, and this data strongly indicated that CaSR might be involved in diabetic cardiac injury. For this regard, we observed the hemodynamic changes and the ratio of apoptosis *in vivo* and *in vitro*, respectively. The present data showed that significant apoptosis and cardiac dysfunction were observed. Furthermore, the apoptosis and cardiac dysfunction could be enhanced or weakened by either CaSR agonist or its antagonist, suggesting the involvement of CaSR in cardiac injury in diabetic rats. In addition, by exposing neonatal cardiomyocytes to 25.5 mM glucose induced significant apoptosis in the preparation, GdCl_3_ exacerbated this injury, whereas NPS-2390 lessened the effect of GdCl_3_. Meanwhile, we observed that hyperglycemia-associated apoptosis was inhibited when the CaSR was silenced. These results further suggested that cell apoptosis in diabetic rat hearts is closely related to the activation of CaSR.

It is well known that diverse factors can induce apoptosis, such as oxidative stress, calcium overload, and cytokines. Other researches have reported that an increase in extracellular calcium or gadolinium could induce a sustained increase in [Ca2+]i in BRL cells in a concentration-dependent manner through the CaSR-PLC-IP3 pathway [19]. In accordance with this observation, we presume that a CaSR-mediated increase in [Ca2+]i plays, at least in part, an important role in apoptosis during diabetic cardiac injury. In our study, we found that [Ca2+]i was significantly elevated in neonatal rat cardiomyocytes treated with high concentration of glucose, and further increased by GdCl3, but attenuated by NPS-2390. When the CaSR was silenced, [Ca2+]i was decreased obviously. These results suggested that CaSR activation could improve intracellular calcium mobilization, consequently lead to calcium overload, and eventually induce diabetic cardiomyocyte apoptosis. These data were also consistent well with our *in vivo* study.

Cumulative evidences indicate that apoptosis pathways include the mitochondrial pathway, the death receptor pathway, and the endoplasmic reticulum stress pathway, and so on. The mitochondrial pathway of apoptosis began with the permeabilisation of the mitochondrial outer membrane [20]. The opening of the permeability transition (PT) pore caused water to enter the mitochondrial matrix, which resulted in rupturing of the outer membrane, further led to the release of apoptogenic proteins including cytochrome C, apoptosis inducing factor (AIF), and endonuclease G [21]–[23]. Released protein cytochrome C activated cytosolic caspase-3, an important effector molecule in apoptosis, through the apoptosis complex formation containing Cyt-c/Apaf-1/caspase-9. However, PT pore independent of mitochondrial membrane permeabilisation is regulated by Bcl-2 family members [24], [25], which can be subdivided into anti-apoptotic members such as Bcl-2 [24] and pro-apoptotic species such as Bax [26]. In our study, high concentration of glucose could up-regulate the expression of Bax, and down-regulate the expression of Bcl-2. The CaSR agonist (GdCl_3_) or antagonist (NPS-2390) could further augment or weaken the effect of glucose. Silencing of CaSR could increase Bcl-2 expression and decrease Bax expression compared with model group. Thus, it was speculated that CaSR activation might lead to apoptosis through mitochondrial pathway associated with the down-regulation of anti-apoptotic protein Bcl-2 and up-regulation of pro-apoptotic protein Bax.

In addition, the mitogen-activated protein kinase (MAPK) signal pathway is essential for the regulation of proliferation, differentiation, and apoptosis. In mammalian cells, MAPK has been classified into at least three subfamilies [27]: ERK group, JNK/SAPK group, and p38 MAPK. A previous study showed that the stimulation of CaSR was associated with P-ERK1/2 in acutely-dispersed bovine parathyroid cells and human embryo kidney cells [19]. The ERK pathway plays a pivotal role in regulating cell growth and differentiation [28], both by inhibiting components of the cell death machinery and increasing the transcription of pro-survival genes. JNK is an important branch of MAPK pathway, and mediates various cellular responses including programmed cell death (apoptosis), epithelial sheet movement, and planar polarity [29]. Activation of the JNK pathway is required for the release of cytochrome C from the mitochondria and the subsequent activation of the caspase cascade. Therefore, abrogation of the JNK signaling pathway causes various defects in developmental or stress induced apoptosis. The p38 MAPK is also an important branch of MAPK pathway that was originally found to inhibit inflammatory cytokine production and cell death following cellular stress [30]. Recently, it has also been proven that p38 was involved in nitric oxide-induced apoptosis in neurons [31].

In order to confirm the role of MAPK pathway in apoptosis caused by high concentration of glucose, and to validate the relationship between MAPK and CaSR activation, the present study focused on the ERK, JNK, and p38 pathways. We found that high concentration of glucose, GdCl3 and NPS-2390 had no influence on total ERK1/2, JNK, and p38 expression. But we observed the increases in the levels of P-ERK1/2 and P-JNK by GdCl3, and these increases could be reduced by NPS-2390. When we used the siRNA technique to silence CaSR, P-ERK1/2 and P-JNK expressions were decreased compared with model group. Clearly, the modulation of apoptosis is a complex process. All of the involving signal molecules are integrated into a single coherent network, and the quantities of pro-apoptotic and anti-apoptotic factors in the network are related to cell fate. Our data have shown that the activation of CaSR enhanced [Ca2+]i, leading to the release of apoptosis promoters and stimulating apoptotic enzymes. It also was demonstrated that the activation of CaSR could increase P-ERK to confer protection against apoptosis and also to activate P-JNK to promote apoptosis. The dynamic balance between the activities of these pathways is critical in determining the final cellular outcome after high concentration of glucose injury. Our study demonstrated that the pro-apoptotic role of CaSR was dominant during treatment with high concentration of glucose in neonatal rat cardiomyocytes.

The study by Bai et al [32] demonstrated that CaSR expression was decreased in myocardium of diabetes mellitus rats. However, in the present study, we detected that the expression of CaSR was increased in hearts of STZ-diabetic rats. This discrepancy might be due to the difference of diabetic models. In Bai's study, the model was established by intraperitoneal injection of STZ (30 mg/kg) after high-fat and high-sugar diet for one month, which was the type 2 diabetes mellitus. In our experiment, the model was induced in rats with a single intraperitoneal injection of 40 mg/kg STZ, which was the type 1 diabetes mellitus. Thus, based on the results obtained, we proposed that CaSR expression was different in type 1 and type 2 diabetes mellitus, but more detailed studies should be done.

Taken all data together, CaSR activation could lead to the apoptosis of cardiomyocytes in simulated diabetic cardiac injury through the induction of calcium overload and the activation of the mitochondrial and mitogen-activated protein kinase pathway. These findings may serve as potential therapeutic and drug targets.
