# Expression of Concern: Crucial Role of Calcium-Sensing Receptor Activation in Cardiac Injury of Diabetic Rats

**DOI:** 10.1371/journal.pone.0286128

**Published:** 2023-05-18

**Authors:** The PLOS ONE Editors

Following the publication of this article [1], concerns were raised regarding the results presented in Figures 8 and 9. Specifically,

The following panels appear similar:

- Figure 8C Bax and Figure 9A P-ERK
- Figure 8B GAPDH and Figure 8C GAPDH
- Figure 9A T-ERK and Figure 9C T-JNK
- Figure 9B P-ERK and Figure 9D P-JNK

The corresponding author stated that some panels were accidentally duplicated during figure preparation and provided replacement figures as well as underlying data. The individual level data underlying Figures 2, 8, and 9, as well as representative data underlying Figures 8B, 8C, and 9A-D are provided in the S1 and S2 Files below. However, the authors did not agree to publicly share the raw blots underlying the Figure 8 and 9 results, as these data also included results belonging to other researchers. Regarding these data, the corresponding author explained that they re-analysed the western blot data using image J software and created new bar charts for Figures 2, 8, and 9, by calculating the mean ratio of the target band/internal control using 6–8 samples for each group.

The editorial assessment of the provided underlying data, including data not shared with this notice, raised additional concerns that some of the repeat experiment data did not appear to consistently support the published Figure 8 and Fig 9 results, and that differences in reported intensity appeared to be the result of differences in lane width as opposed to differences in signal intensity. The corresponding author did not agree with the editorial assessment and expressed that the differences may be due to differences in loading volume, and differences in exposure time.

The editorial team also noted that the results for the proteins of interest and their respective controls appear to have been obtained from different gels, which was confirmed by the corresponding author. Therefore, the reported controls cannot be considered as internal controls for protein loading and transfer.

The above issues remain unresolved as of the time of this notice and call into question the reliability of results reported for Figures 8 and 9.

The authors are working with PLOS to try and address these issues. Meanwhile, *PLOS ONE* Editors issue this Expression of Concern to notify readers of the above issues, and to relay the supporting data provided by the corresponding author.

## Supporting information

S1 File Individual level data underlying Figures 2, 8, and 9. (ZIP)

S2 File Representative data underlying the published Figures 8B, 8C, 9A-D results. (PDF)
