# Supplementary material for: Expression of Concern: Crucial Role of Calcium-Sensing Receptor Activation in Cardiac Injury of Diabetic Rats
Source: PLoS One. 2023 May 18;18(5):e0286128. doi: 10.1371/journal.pone.0286128 (PMC10194876; doi:10.1371/journal.pone.0286128)
Supplement: S2 File — (PDF) [file pone.0286128.s002.pdf]

Figure 8B

Representative figure

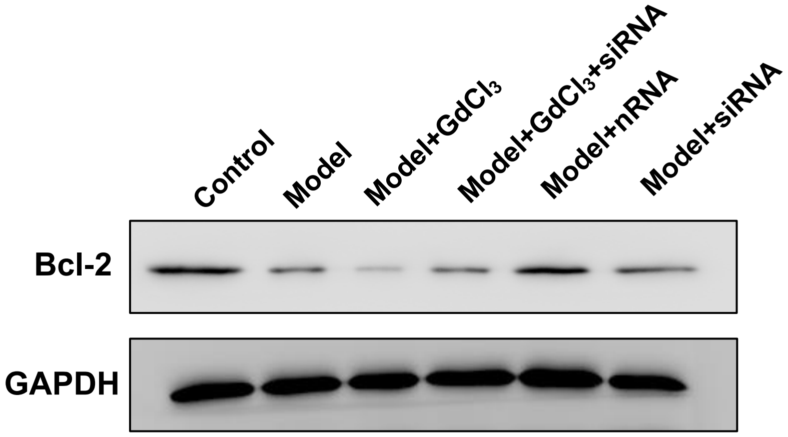

Group	Control	Model	Model+$GdCl_3$	Model+$GdCl_3$+siRNA	Model+nRNA	Model+siRNA
Bcl-2	32696.095	11514.054	6952.205	13920.418	23681.489	17717.903
GAPDH	26042.539	25463.983	20759.69	27938.104	29192.276	21757.882

1

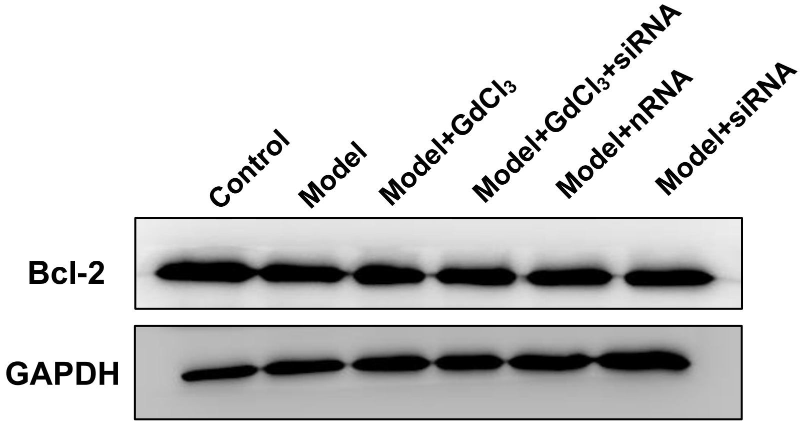

Group	Control	Model	Model+$GdCl_3$	Model+$GdCl_3$+siRNA	Model+nRNA	Model+siRNA
Bcl-2	31596.903	25719.518	17695.69	23973.69	25344.468	25306.004
GAPDH	18810.175	26952.69	27083.518	22128.326	26510.69	32197.246

2

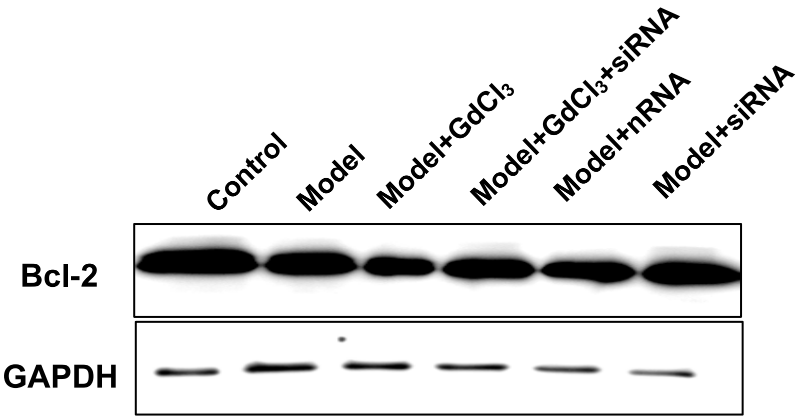

Group	Control	Model	Model+$GdCl_3$	Model+$GdCl_3$+siRNA	Model+nRNA	Model+siRNA
Bcl-2	34488.974	23980.054	9624.447	19411.518	19217.539	23417.539
GAPDH	18295.447	20937.225	16313.69	16130.104	13836.326	13089.74

3

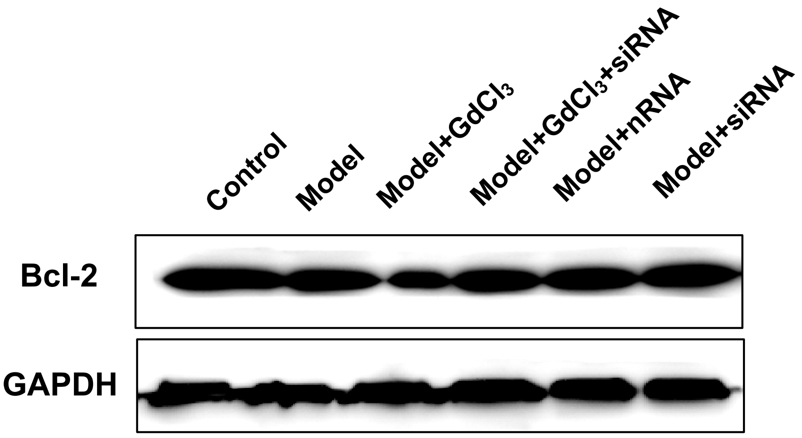

Group	Control	Model	Model+$GdCl_3$	Model+$GdCl_3$+siRNA	Model+nRNA	Model+siRNA
Bcl-2	34192.317	26501.589	12394.719	25770.347	27123.882	29685.418
GAPDH	23778.175	26823.983	30464.761	29225.61	25650.004	26013.418

4

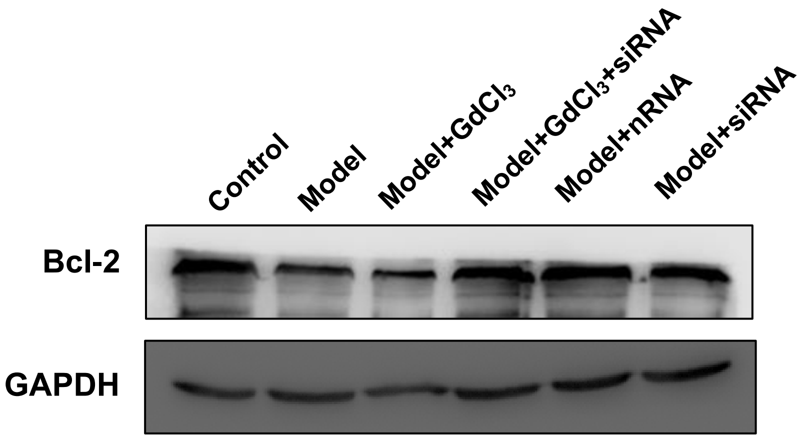

Group	Control	Model	Model+$GdCl_3$	Model+$GdCl_3$+siRNA	Model+nRNA	Model+siRNA
Bcl-2	27049.296	15418.518	9979.983	26356.882	28648.589	22805.468
GAPDH	17010.296	18746.539	15716.418	24511.418	26491.882	27747.024

5

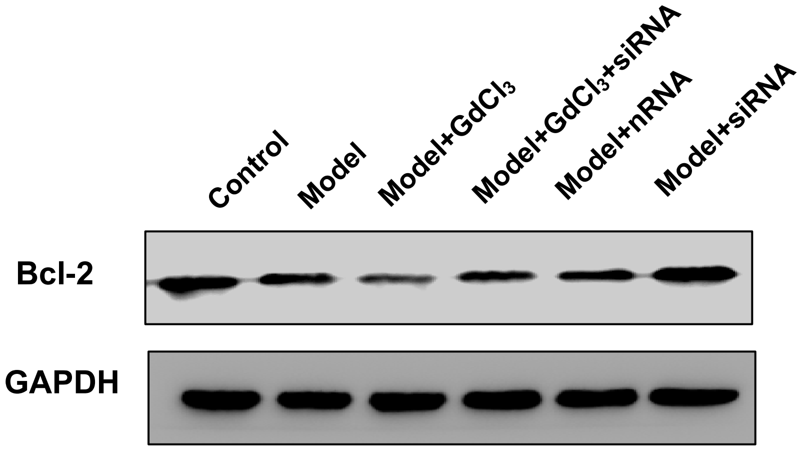

Group	Control	Model	Model+$GdCl_3$	Model+$GdCl_3$+siRNA	Model+nRNA	Model+siRNA
Bcl-2	23470.246	13133.054	7864.861	14848.276	13706.397	23549.832
GAPDH	20369.539	22328.397	24841.104	21866.69	23009.104	27884.418

Figure 8C

Representative figure

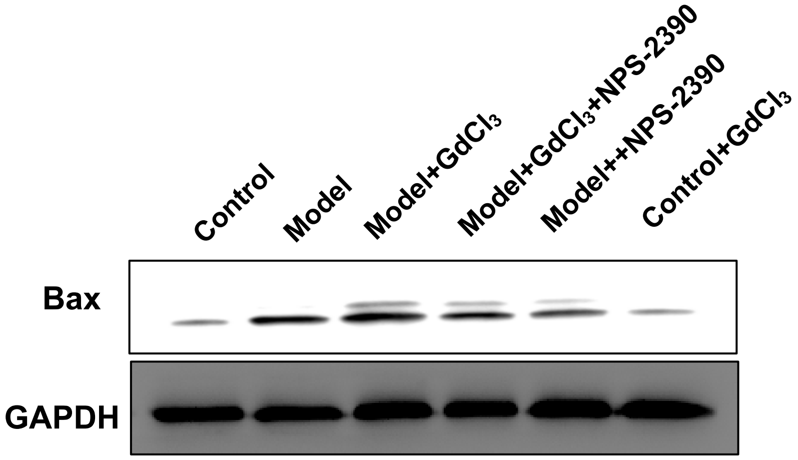

Group	Control	Model	Model+$GdCl_3$	Model+$GdCl_3$+NPS-2390	Model+NPS-2390	Control+$GdCl_3$
Bax	7341.669	17374.397	26738.246	13816.983	12163.811	9821.255
GAPDH	23382.196	24474.004	23609.054	22789.376	25425.69	23796.782

1

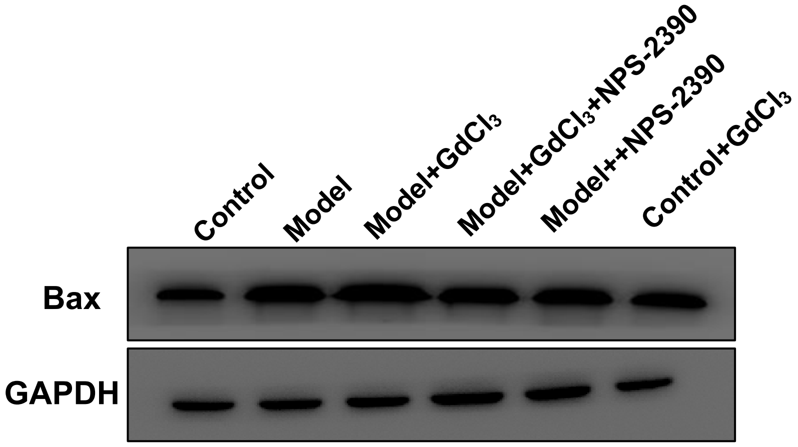

Group	Control	Model	Model+$GdCl_3$	Model+$GdCl_3$+NPS-2390	Model+NPS-2390	Control+$GdCl_3$
Bax	15420.518	28326.861	34901.276	26722.447	27402.983	20697.225
GAPDH	22699.418	21150.125	20639.761	27795.782	25560.782	17460.296

2

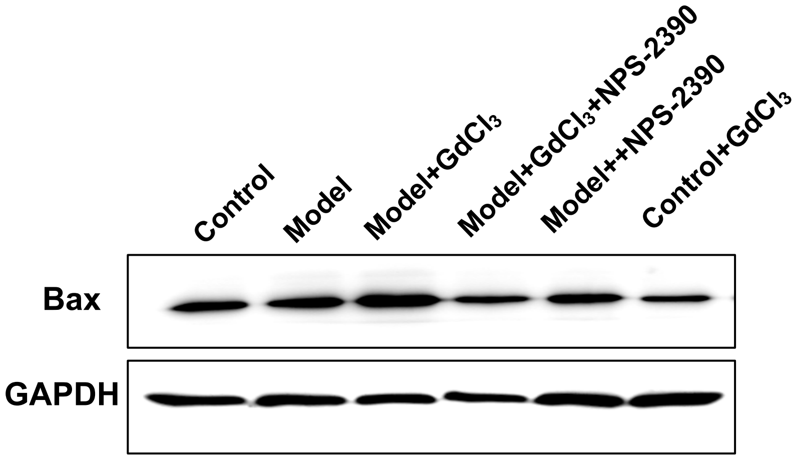

Group	Control	Model	Model+$GdCl_3$	Model+$GdCl_3$+NPS-2390	Model+NPS-2390	Control+$GdCl_3$
Bax	16186.589	21295.589	29526.296	15051.64	19407.933	18442.326
GAPDH	26342.832	24487.296	17761.79	17389.276	26831.518	26273.69

3

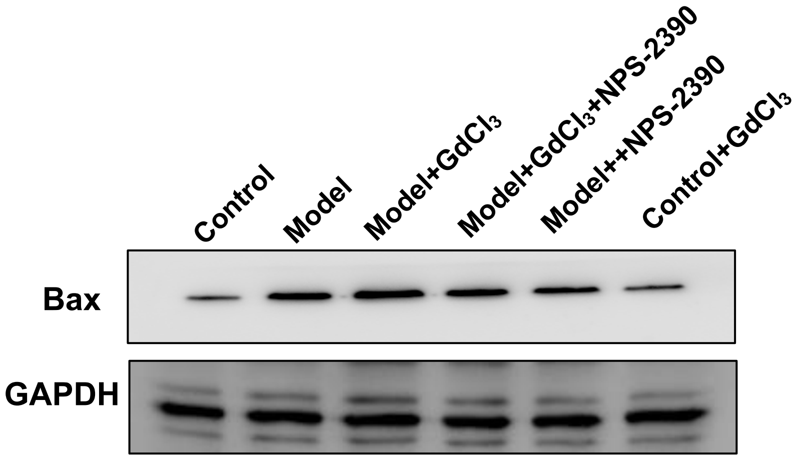

Group	Control	Model	Model+$GdCl_3$	Model+$GdCl_3$+NPS-2390	Model+NPS-2390	Control+$GdCl_3$
Bax	11453.175	19352.296	24899.296	19454.711	21807.64	13428.125
GAPDH	24633.61	21338.518	21901.054	24148.711	24297.246	14952.64

4

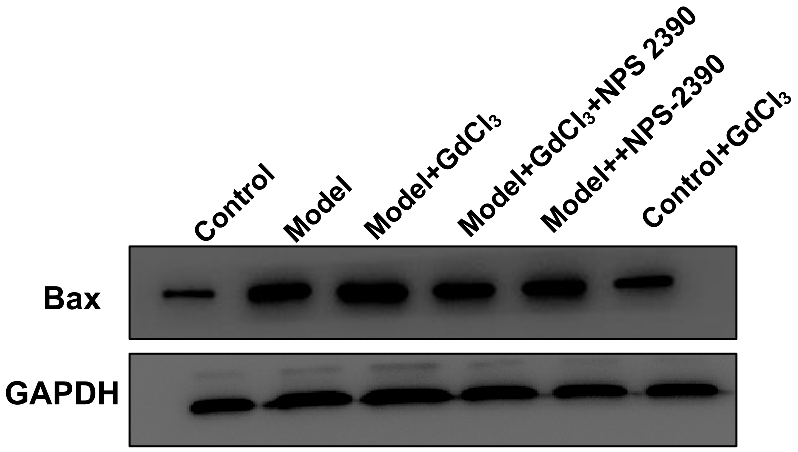

Group	Control	Model	Model+$GdCl_3$	Model+$GdCl_3$+NPS-2390	Model+NPS-2390	Control+$GdCl_3$
Bax	7230.438	25687.075	32219.004	25868.832	25395.731	11251.74
GAPDH	13698.711	26300.125	26639.589	26021.711	26479.711	14359.64

5

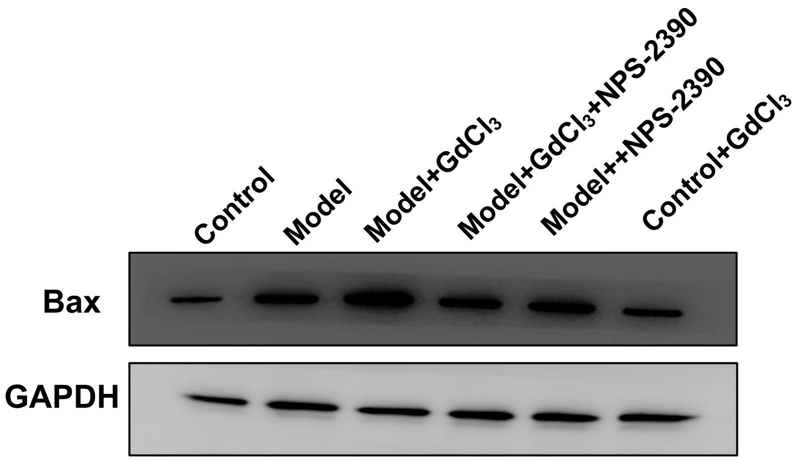

Group	Control	Model	Model+$GdCl_3$	Model+$GdCl_3$+NPS-2390	Model+NPS-2390	Control+$GdCl_3$
Bax	11589.296	21764.539	28871.468	21344.196	22026.175	14988.657
GAPDH	17827.903	23527.589	22453.054	22062.468	22200.125	18444.882

Figure 9A

Representative figure

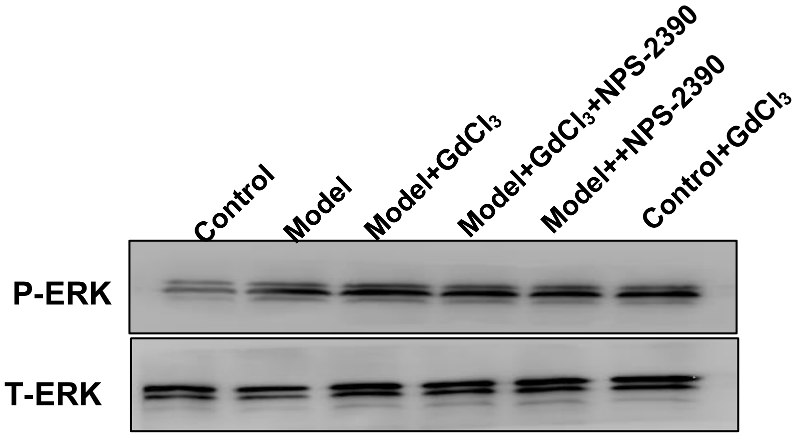

Group	Control	Model	Model+$GdCl_3$	Model+$GdCl_3$+NPS-2390	Model+NPS-2390	Control+$GdCl_3$
P-ERK	11640.882	23629.125	31591.418	27316.933	25348.347	27759.539
T-ERK	20515.711	17059.397	23615.175	24083.711	26399.882	27106.418

1

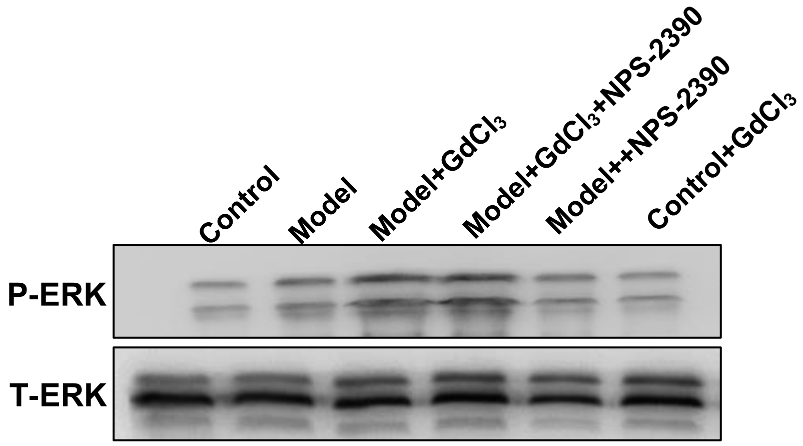

Group	Control	Model	Model+$GdCl_3$	Model+$GdCl_3$+NPS-2390	Model+NPS-2390	Control+$GdCl_3$
P-ERK	9772.518	15662.69	28767.196	26813.66	13427.225	13213.619
T-ERK	28986.882	26943.447	26974.347	27564.104	28370.589	25078.004

2

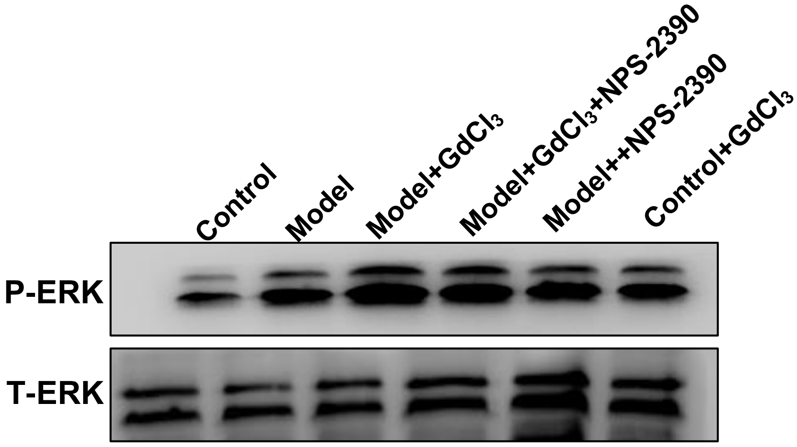

Group	Control	Model	Model+$GdCl_3$	Model+$GdCl_3$+NPS-2390	Model+NPS-2390	Control+$GdCl_3$
P-ERK	10753.418	19815.882	35472.485	21752.761	21750.175	20308.246
T-ERK	17161.589	14611.054	13726.468	23718.054	24089.296	19322.054

3

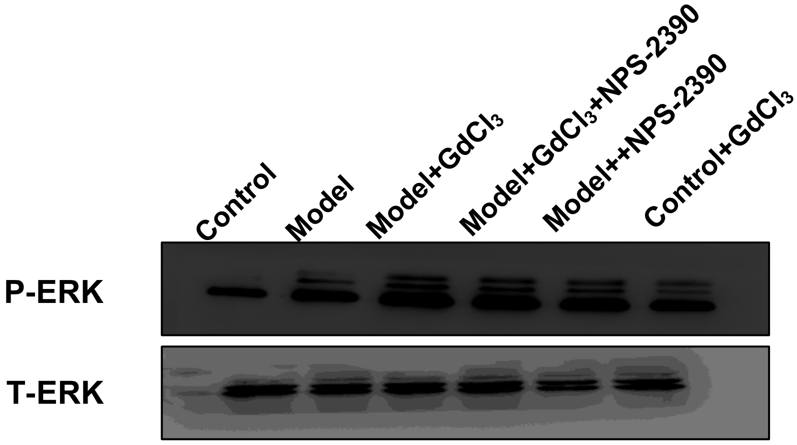

Group	Control	Model	Model+$GdCl_3$	Model+$GdCl_3$+NPS-2390	Model+NPS-2390	Control+$GdCl_3$
P-ERK	9014.861	16559.953	32432.418	24482.418	22961.125	20987.953
T-ERK	29291.66	25938.276	26481.69	28953.861	22195.811	25362.489

4

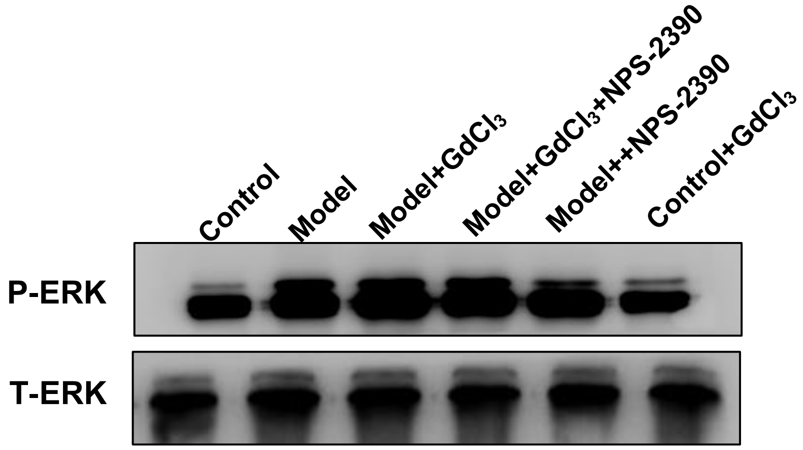

Group	Control	Model	Model+$GdCl_3$	Model+$GdCl_3$+NPS-2390	Model+NPS-2390	Control+$GdCl_3$
P-ERK	15367.276	25498.619	30211.69	25999.154	26254.933	18675.347
T-ERK	23571.589	23406.64	23792.468	24776.397	24060.832	20732.347

5

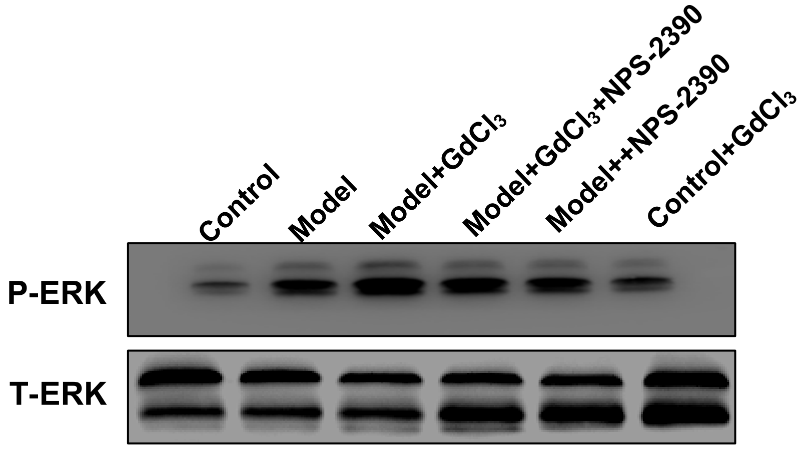

Group	Control	Model	Model+$GdCl_3$	Model+$GdCl_3$+NPS-2390	Model+NPS-2390	Control+$GdCl_3$
P-ERK	8410.418	19851.933	27676.933	22464.569	19660.054	17072.832
T-ERK	21546.276	18430.719	17830.64	22519.569	21913.104	23033.104

Figure 9B

Representative figure

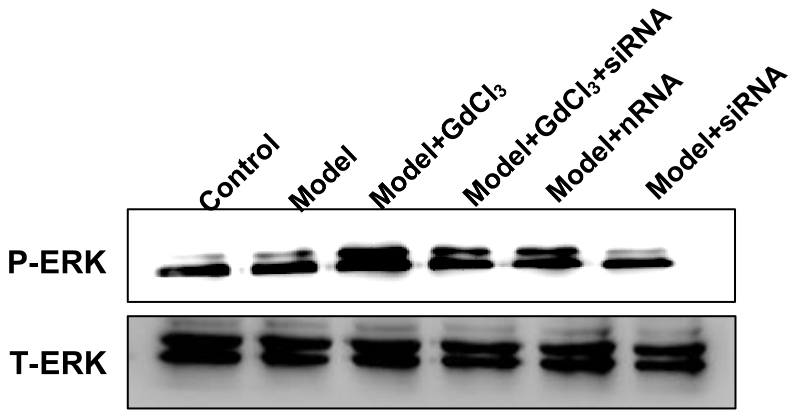

Group	Control	Model	Model+$GdCl_3$	Model+$GdCl_3$+siRNA	Model+nRNA	Model+siRNA
P-ERK	11217.225	14196.397	25200.175	16559.104	17710.125	11271.569
T-ERK	25280.418	19744.468	19627.104	19747.418	22009.368	17682.64

1

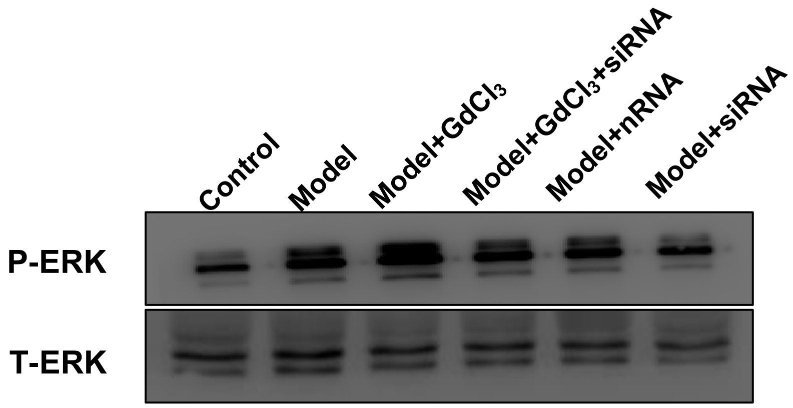

Group	Control	Model	Model+$GdCl_3$	Model+$GdCl_3$+siRNA	Model+nRNA	Model+siRNA
P-ERK	10637.983	20142.711	25719.004	17923.054	16989.296	11366.518
T-ERK	23143.903	25528.66	19978.175	19241.539	17940.418	14985.64

2

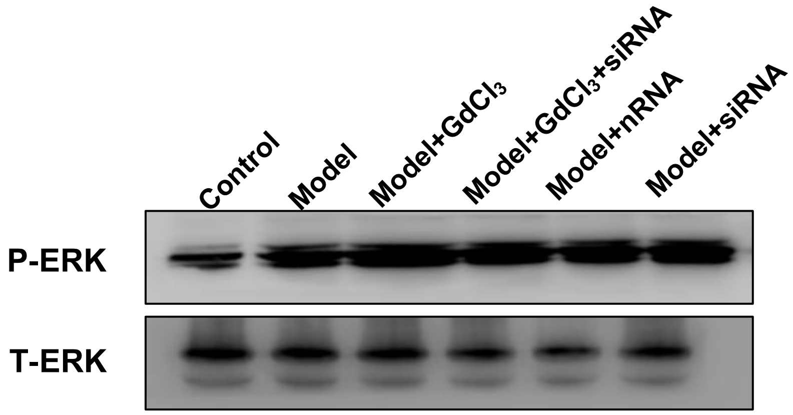

Group	Control	Model	Model+$GdCl_3$	Model+$GdCl_3$+siRNA	Model+nRNA	Model+siRNA
P-ERK	13959.296	24563.104	37294.64	29990.326	27546.033	29778.569
T-ERK	25438.489	23922.468	24712.246	19881.125	16479.347	21296.731

3

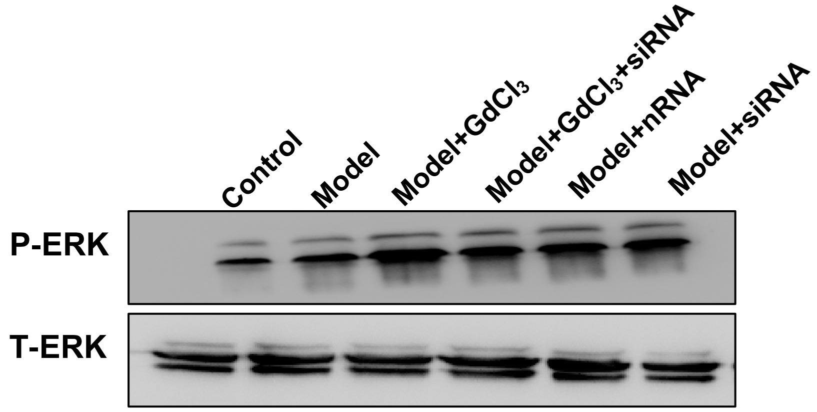

Group	Control	Model	Model+$GdCl_3$	Model+$GdCl_3$+siRNA	Model+nRNA	Model+siRNA
P-ERK	9786.225	16384.518	28863.761	22146.569	25368.175	23538.832
T-ERK	20462.246	21684.125	21953.468	27009.024	21130.882	17876.175

4

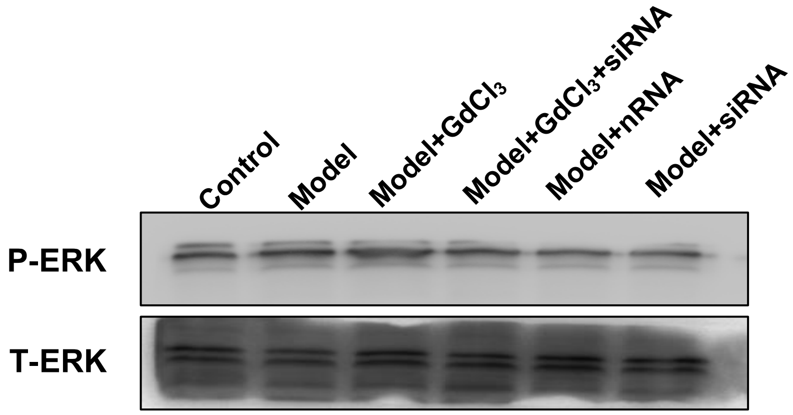

Group	Control	Model	Model+$GdCl_3$	Model+$GdCl_3$+siRNA	Model+nRNA	Model+siRNA
P-ERK	20272.225	27430.652	30935.782	22082.418	16586.347	16545.418
T-ERK	27557.803	28763.69	27166.569	28791.276	23778.326	29072.539

5

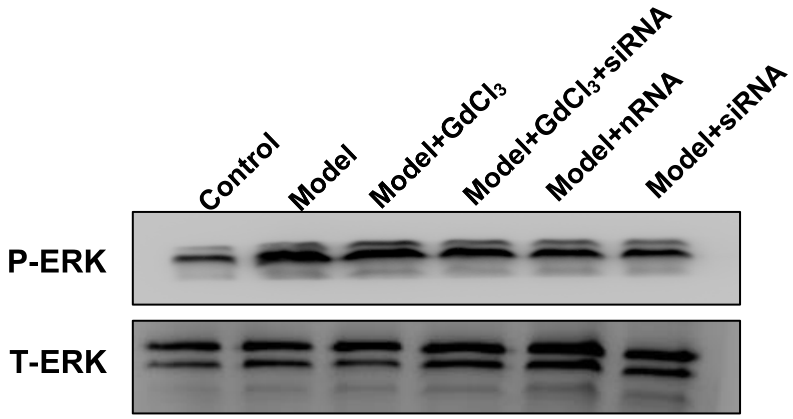

Group	Control	Model	Model+$GdCl_3$	Model+$GdCl_3$+siRNA	Model+nRNA	Model+siRNA
P-ERK	11278.861	26649.125	28731.761	23873.347	21980.711	18992.589
T-ERK	13328.882	17092.347	16590.347	23581.468	25263.347	20989.882

Figure 9C

Representative figure

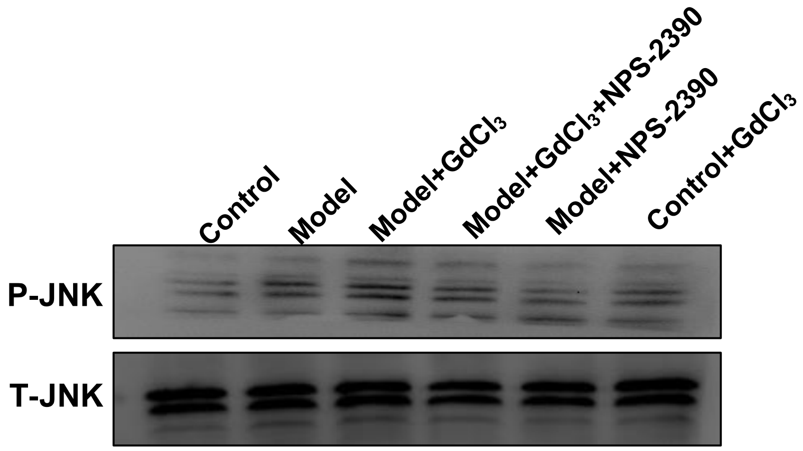

Group	Control	Model	Model+$GdCl_3$	Model+$GdCl_3$+NPS-2390	Model+NPS-2390	Control+$GdCl_3$
P-JNK	12234.731	20323.761	23776.004	19559.64	17702.832	25307.631
T-JNK	26676.246	25079.518	26962.589	21838.69	24384.711	28196.418

1

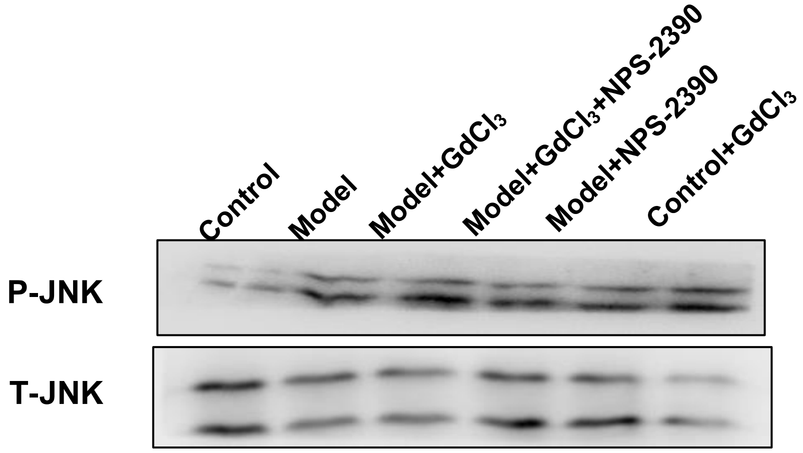

Group	Control	Model	Model+$GdCl_3$	Model+$GdCl_3$+NPS-2390	Model+NPS-2390	Control+$GdCl_3$
P-JNK	7947.075	21793.246	26585.782	21994.125	23394.004	25387.539
T-JNK	24906.711	19677.933	18424.175	24270.246	23325.711	17840.832

2

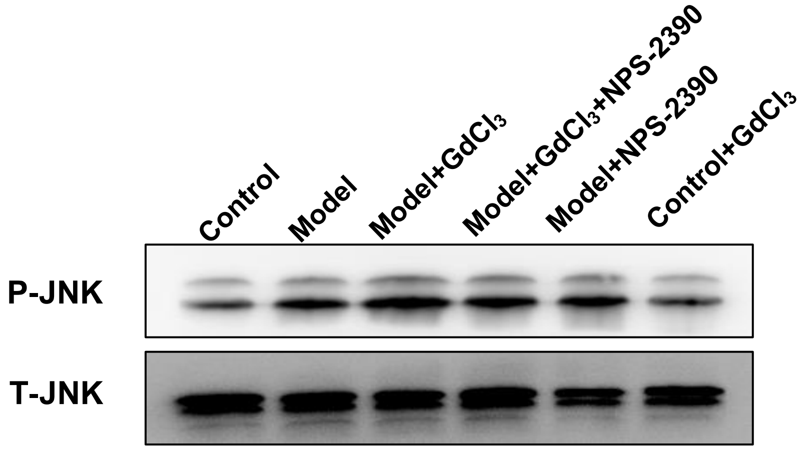

Group	Control	Model	Model+$GdCl_3$	Model+$GdCl_3$+NPS-2390	Model+NPS-2390	Control+$GdCl_3$
P-JNK	10748.761	18862.711	28244.61	21917.711	20891.418	13951.125
T-JNK	29877.388	26531.175	24608.518	27597.589	19126.711	22508.66

3

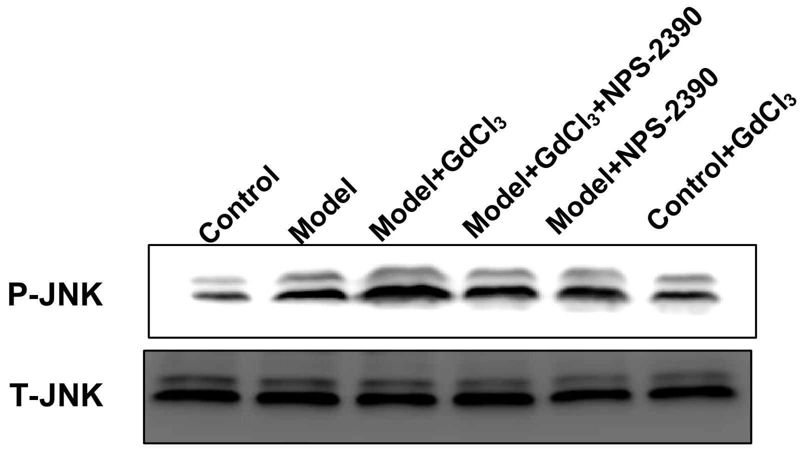

Group	Control	Model	Model+$GdCl_3$	Model+$GdCl_3$+NPS-2390	Model+NPS-2390	Control+$GdCl_3$
P-JNK	8550.083	16486.933	30124.368	19618.004	18446.468	12476.104
T-JNK	23663.196	20869.711	25469.296	23800.004	26091.418	25096.004

4

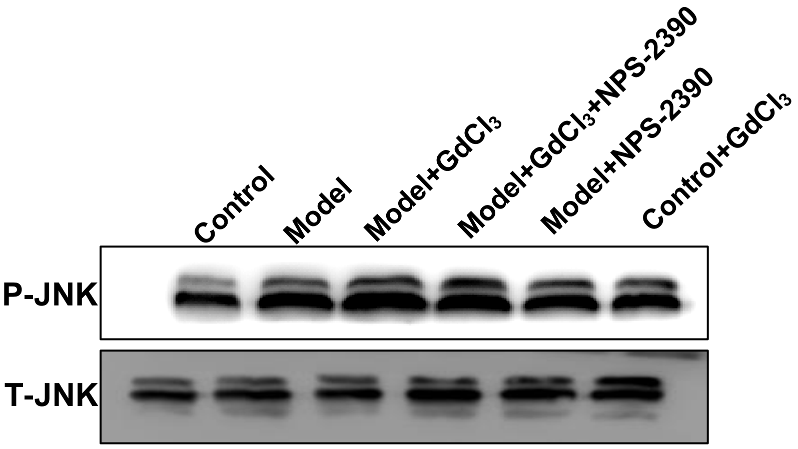

Group	Control	Model	Model+$GdCl_3$	Model+$GdCl_3$+NPS-2390	Model+NPS-2390	Control+$GdCl_3$
P-JNK	12147.769	25609.811	31092.125	23867.104	22252.69	20061.933
T-JNK	18794.539	22931.711	18308.368	22245.296	21093.368	18469.64

5

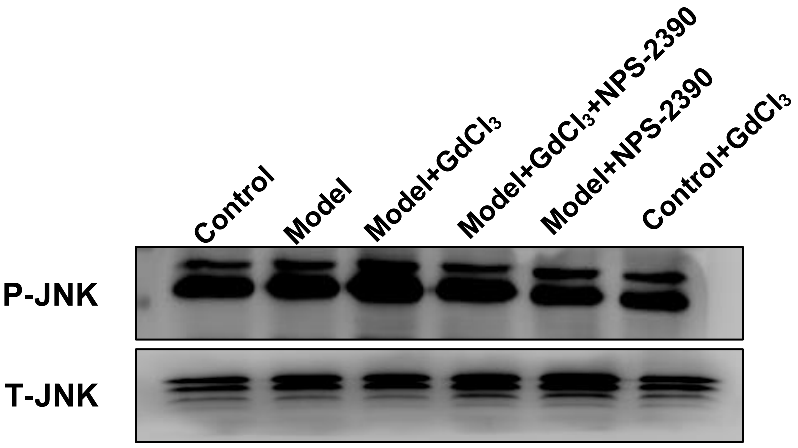

Group	Control	Model	Model+$GdCl_3$	Model+$GdCl_3$+NPS-2390	Model+NPS-2390	Control+$GdCl_3$
P-JNK	21621.882	22807.64	27089.74	23407.811	20089.64	19716.347
T-JNK	24715.711	24568.033	23625.154	26817.861	30262.983	22132.66

Figure 9D

Representative figure

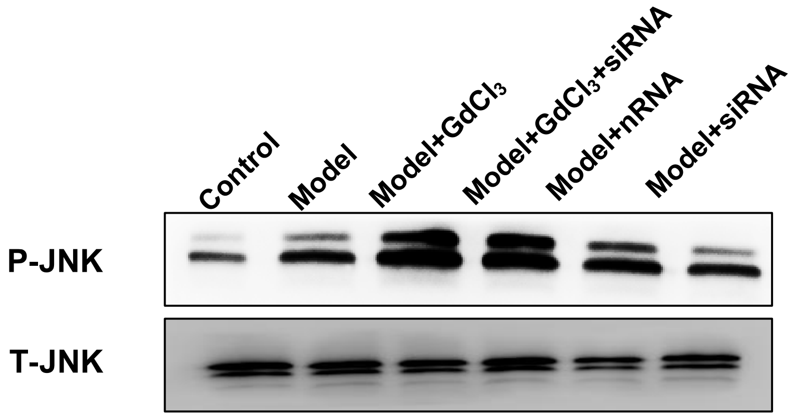

Group	Control	Model	Model+$GdCl_3$	Model+$GdCl_3$+siRNA	Model+nRNA	Model+siRNA
P-JNK	4578.841	11550.569	28624.196	20846.154	15428.811	9985.619
T-JNK	30623.681	26639.861	22226.69	26213.518	18473.589	23935.418

1

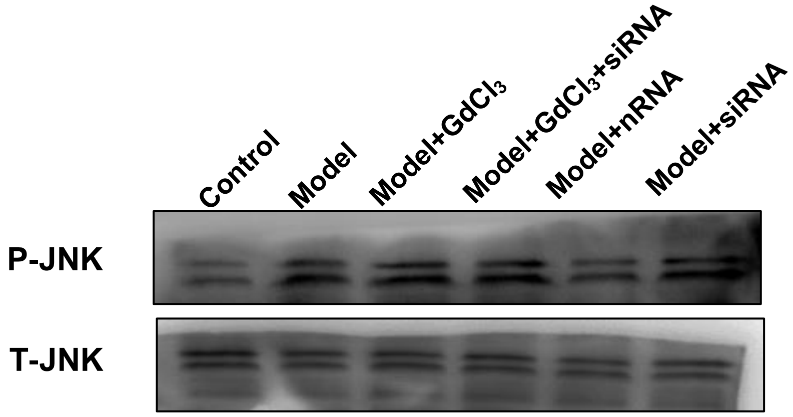

Group	Control	Model	Model+$GdCl_3$	Model+$GdCl_3$+siRNA	Model+nRNA	Model+siRNA
P-JNK	8326.054	15166.368	18300.075	18040.832	13633.104	30314.196
T-JNK	30344.711	22231.054	22909.882	28012.589	22612.125	22034.246

2

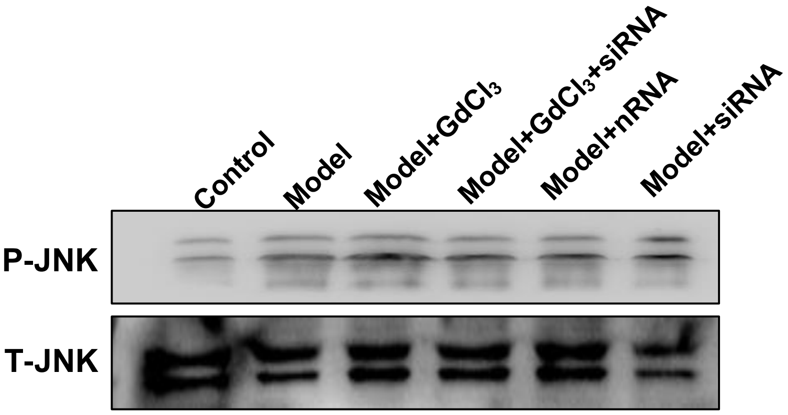

Group	Control	Model	Model+$GdCl_3$	Model+$GdCl_3$+siRNA	Model+nRNA	Model+siRNA
P-JNK	6018.296	19884.953	31049.024	19079.882	20831.125	18627.175
T-JNK	25519.125	21232.711	21042.075	24358.347	25866.276	17759.095

3

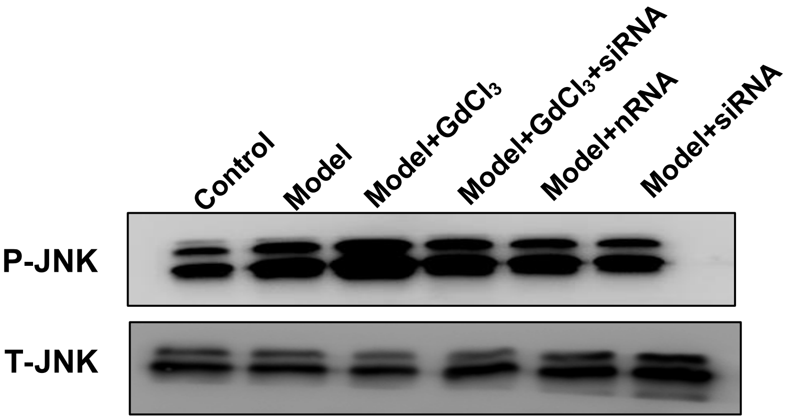

Group	Control	Model	Model+$GdCl_3$	Model+$GdCl_3$+siRNA	Model+nRNA	Model+siRNA
P-JNK	12660.078	25751.397	35335.205	23835.912	25107.569	21400.296
T-JNK	24427.861	23196.296	17202.033	17322.276	25193.811	26500.711

4

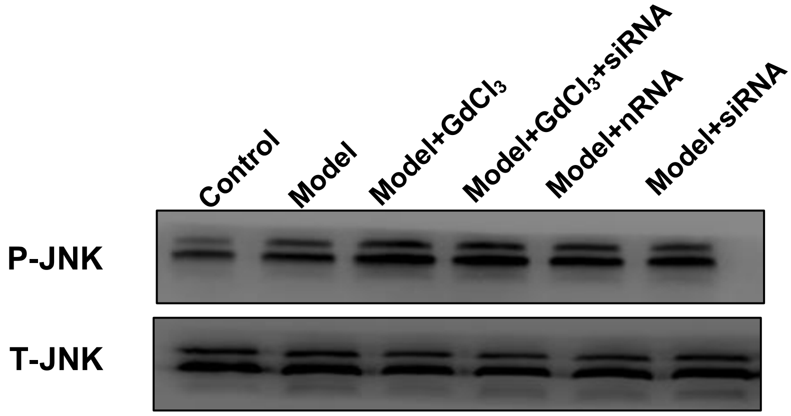

Group	Control	Model	Model+$GdCl_3$	Model+$GdCl_3$+siRNA	Model+nRNA	Model+siRNA
P-JNK	12306.347	20324.539	32124.953	22729.418	21681.589	18471.882
T-JNK	31624.518	26444.296	24952.296	25069.619	30468.468	27112.832

5

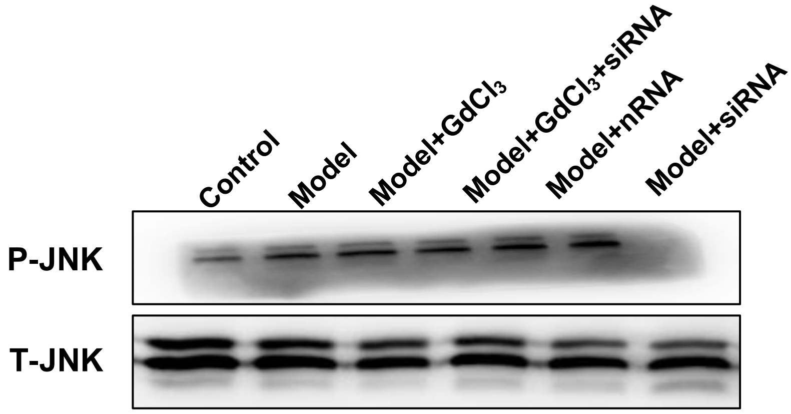

Group	Control	Model	Model+$GdCl_3$	Model+$GdCl_3$+siRNA	Model+nRNA	Model+siRNA
P-JNK	14358.246	22523.397	28536.276	22871.276	34079.468	28781.397
T-JNK	29559.397	21006.933	21545.64	19908.347	27075.832	29757.518
